# England's Dementia Diagnosis Landscape: Strengths, Limitations, and Future Directions

**DOI:** 10.1002/gps.70236

**Published:** 2026-07-02

**Authors:** Jemma Hazan, Robert Howard, Jeremy D. Isaacs

**Affiliations:** ^1^ Division of Psychiatry University College London London UK; ^2^ North London NHS Foundation Trust London UK; ^3^ St George's University Hospitals NHS Foundation Trust London UK; ^4^ Department of Psychology & Neuroscience School of Health & Medical Sciences City St George's University of London London UK

## Abstract

**Background:**

The National Health Service (NHS) England Primary Care Dementia Data (PCDD) provides a comprehensive dementia registry, underpinning national policy to improve diagnosis, care, and support.

**Methods:**

This paper evaluates the PCDD in comparison with international quality dementia registries (SveDem, NorCog, ADNeT).

**Results:**

We highlight strengths in PCDD coverage and case ascertainment, but also weaknesses in diagnostic specificity and post‐diagnostic metrics.

**Conclusions:**

We review currently collected metrics and discuss potential data expansions, including dementia severity and NICE‐approved therapy uptake, alongside new targets, such as an 18‐week standard for memory assessment service referrals.

## Key Points

- The NHS England PCDD provides comprehensive national coverage. Compared with international registries it captures less information on dementia subtype.
- Future data collection could include dementia severity measures and the use of NICE‐approved dementia pharmacotherapies to enhance quality improvement and policy evaluation.

## 1 Introduction

The National Health Service (NHS) England Primary Care Dementia Data [1] (NHS England PCDD), is a monthly publication reporting on a range of metrics associated with dementia diagnosis and care. The dataset reflects and has informed policy, notably a dementia diagnosis rate (DDR) target (two‐thirds of people in England with dementia should have a diagnosis), and highlighted regional variations in the DDR and other parameters [2]. Notably, England is one of only eight countries that estimate the prevalence of people with dementia [3].

In epidemiology, a *register* is a comprehensive dataset of cases of a specific disease within a defined population, while a *registry* is an ongoing system for continuously collecting and managing such data [4]. Under this definition, the NHS England PCDD qualifies as a national data registry, systematically gathering data sourced from general practice (GP) records across England. However, it has not been formally classified as a *quality registry* [5], a term used in some countries to describe registries specifically established to support quality improvement, benchmarking and outcomes monitoring through standardised data collection. For example, the Swedish Registry for Cognitive and Dementia Disorders (SveDem) was established in 2007 as a leading national quality registry for dementia [6]. Despite this distinction the PCDD shares many of the objectives of quality registries, including improving patient care, providing quality assurance, monitoring indicators and informing long‐term policy and research [7].

With a focus on diagnosis, this commentary evaluates the NHS dataset currently collected metrics, makes comparisons with international quality registries and proposes expansions to align with global quality registry standards.

## 2 Current Data Metrics

Comparing the NHS England PCDD with international registries requires an understanding of their distinct contexts and purposes. We compared the PCDD with quality registries that provide publicly available annual aggregate data reports. Registries were identified through a targeted literature search and review of national registry reports. Sweden's SveDem, the Norwegian Registry of Persons Assessed for Cognitive Symptoms (NorCog) [8, 9], and Australia's Dementia Network Registry (ADNeT) were selected as they are established national registries with comparable reported diagnostic and quality‐of‐care metrics [10]. Data were extracted on registry structure, data sources, coverage, participation model, and key metrics including diagnosis proportions and antipsychotic prescribing (Table 1). All data were aggregate and publicly available.

**TABLE 1:** Comparison of dementia registry metrics: Diagnosis, subtyping, and treatment.

Country	Registry name	Data sources	Coverage	Report	Key metrics	Notes
Sweden	SveDem (The Swedish registry for cognitive and dementia disorders)	Memory clinics, primary care, hospitals, special residential care (SÄBO), home care (HEMO)	57 specialist care units+ 926 primary care units 2023 new participants: 5733 Total participants (2007–2023): 121,334	2023 annual report [8]	− Annual proportion of dementia diagnoses: 79% (4514/5733) − Annual proportion of MCI diagnoses: Not reported − Annual proportion of unspecified dementia diagnoses: 21% (948/4514 dementia cases) − Annual antipsychotic prescribing proportion in dementia cases: 4.4% (SÄBO module, 254/5772)	Fully centralised, integrates clinic, primary care, and residential care data. Voluntary (opt‐in for patients). Antipsychotic data specific to SÄBO module
Australia	Australian dementia network (ADNeT) registry	Memory clinics	82 sites (26% regional, 43% private) 2024 new participants: 2619 Total participants (2020–2024): 6898	2024 annual report [10]	− Annual proportion of dementia diagnoses: 68% (1774/2619) − Annual proportion of MCI diagnoses: 32% (845/2619) − Annual proportion of unspecified dementia diagnoses: Not reported (2% in 2023 report) − Annual antipsychotic prescribing proportion in dementia cases: 6% (106/1774)	Voluntary (opt‐in for patients). Centralized, managed by monash university. 2023 report confirms 2% unspecified dementia; 2024 lacks this detail.
Norway	NorCog (Norwegian registry of persons assessed for cognitive symptoms)	Geriatric, old‐age psychiatric, and memory clinics	70% coverage (for dementia diagnoses, compared to Norwegian patient registry, 2024) 82 sites 2024 new participants: 2661 Total participants (2008–2024): 27,459	2024 annual report [9]	− Annual proportion of dementia diagnoses: 43% (1154/2661) − Annual proportion of MCI diagnoses: 41% (1091/2661) − Annual proportion of unspecified dementia diagnoses: 15% (173/1154) − Annual antipsychotic prescribing proportion in dementia cases: Not reported	Voluntary (consent‐based, transitioning to opt‐out in 2025). Centralised, managed by national centre for ageing and health and Oslo University hospital.
England	NHS primary care dementia data	GP practices	98.67% practice coverage (6138/6221 practices, July 2025) 2025 participants: 506,549 (dementia register) MCI: 219,370 (40+ without dementia)	September 2025 data [29]	− Annual proportion of dementia diagnoses: Not reported (total register: 510,536). Dementia diagnosis rate 65+ = 66.3% − Annual proportion of MCI diagnoses: Not reported (total MCI: 226,597) − Annual proportion of unspecified dementia diagnoses: 28.2% (143,831/510,536) − Annual antipsychotic prescribing proportion in dementia cases: 8.7% (44,660/510,536; 9443 with psychosis, 35,217 without)	Centralised via NHS england, mandatory data collection from GP practices.
England	National audit of dementia spotlight audit in memory assessment services (NAD‐MAS)	Memory clinics	6148 records, 125 services, 10–96 records per service, 2 services excluded with < 25 records, targeting 40+ patients	2023 audit report [11]	− Dementia diagnosis proportion: 71% (4335/6148) − MCI diagnosis proportion: 17% (1045/6148) − Unspecified dementia proportion: Not reported − Antipsychotic prescribing proportion: Not reported	Case note audit, centralised by royal college of psychiatrists.

The PCDD provides a population‐level surveillance registry and relies on mandatory GP reporting. This approach ensures near total national coverage of individuals coded with a dementia diagnosis in primary care. A strength of the PCDD is that it is comprehensive; it captures a broad population of prevalent cases across primary care. However, it does not currently link to secondary care datasets, such as hospital or memory clinic data, limiting insights into diagnostic processes and longitudinal outcomes.

In contrast, SveDem integrates primary, specialist, and residential care data, providing a comprehensive longitudinal view, but representing only participating sites. NorCog and ADNeT collect data on patients referred to specialist clinics, thus emphasising incident cases and including cognitive testing and biomarker data; however, voluntary participation by sites reduces representativeness.

These differences, primary versus specialist care, mandatory versus voluntary participation, and total versus new diagnoses, shape metrics such as diagnosis proportions, making direct comparisons complex but revealing strengths and limitations within each system. To enhance comparability with these incident‐focused registries, we incorporated findings from the National Audit of Dementia Spotlight Audit in Memory Assessment Services (NAD‐MAS) 2023 [11], a case note audit of 6148 records submitted by 125 memory services across England.

NHS England PCDD GP practice coverage is extremely high, with 98.65% of 6220 open practices reporting in September 2025, a figure stable across the year and surpassing many other countries. Sweden's SveDem, and Norway's NorCog, approach similarly high levels of completeness, while Australia's ADNeT, spanning 82 memory clinics, is less comprehensive, likely due to its voluntary scope. Although the PCDD reporting is mandatory and coverage is high, data quality remains dependent on the accurate classification and assignment of diagnostic codes and the timely transfer of information to the primary care records.

Dementia diagnoses in England reached an all‐time high of 510,536 across all ages in the September 2025 NHS England PCDD, a 3% increase since September 2024. For those aged 65 and older, diagnoses totalled 495,453 out of an estimated 747,8123 cases, with a diagnosis rate of 66.3%. The NHS England PCDD calculates the dementia diagnosis rate (DDR) by dividing the number of people with a recorded dementia diagnoses in primary care (the numerator) by the modelled prevalence estimated for the over‐65 population (the denominator), based on epidemiological data from the Cognitive Function and Ageing Study II (CFAS II) [12]. The most recently published DDR was 66.3%, compared to 65.5% 12 months earlier. None of the other national registries report a routinely published national diagnosis rate of this kind. NAD‐MAS 2023 reported that 71% of patients (4335/6148) assessed in memory services received a dementia diagnosis, comparable to SveDem's 79% (4514/5733), ADNeT's 68% (1774/2619), but higher than NorCog's 43% (1154/2661).

Young‐onset dementia (YOD) accounts for 6.8% of NHS England PCDD diagnoses (34,548/510,536), only slightly exceeding global estimates of 5%–6% [13]. This is comparable to SveDem's 6.2% (280/4514) and slightly lower than NorCog's 8.4% (224/2661).

Pathological dementia diagnosis specificity remains a significant challenge. In the NHS England PCDD, Alzheimer's disease (AD) comprises 42.3% of cases (215,800/510,536), well below UK and global estimates of the frequency of AD diagnosis of 60%–70% [14]. Vascular dementia comprised 14.9% of cases (76,190) and mixed dementia 7.9% (41,076), with lower rates for DLB (3.2%) and FTD (0.8%). NAD‐MAS reported that 30% of dementia diagnoses were AD, ranging from 6% to 90% across different services with low rates of dementia with Lewy bodies (DLB) (0%–8%) and frontotemporal dementia (FTD) (0%–5%), with many services reporting no diagnoses. This diagnostic variability could reflect variation in service configuration, expertise or specialist input. In the NHS England PCDD the unspecified dementia proportion is 28.2% (143,831 cases), higher than SveDem's 21%, NorCog's 15%, and ADNeT's 2% (2023), suggesting greater diagnostic imprecision or incomplete coding in primary care. The latter is the more likely explanation, as in NAD‐MAS, the mean unspecified dementia proportion is 5.7%. A possible explanation for loss of diagnostic specificity in the handover of information from secondary to primary care is that memory services generally provide an ICD‐10 diagnostic code, whereas primary care in England uses the Systematised Nomenclature of Medicine Clinical Terms (SNOMED‐CT) coding system. NAD‐MAS reports wide variation in neuroimaging use (0%–90% of patients per site), reflecting resource disparities that may contribute to diagnostic variation.

Mild cognitive impairment (MCI) in the NHS England PCDD was recorded in 226,597 individuals aged 40 and older without dementia in September 2025. The number of estimated MCI diagnoses lags behind dementia diagnoses, likely reflecting less systematic MCI diagnosis and coding, as the prevalence of MCI is thought to exceed that of dementia [15]. NAD‐MAS reported that 17% of patients (1045/6148) were diagnosed with MCI (range 0%–42%; 13 services with no MCI diagnoses). This was lower than NorCog's 41% (1091/2661) and ADNeT's 32% (845/2619), but with a wide range, suggesting heterogenous referral and clinical practices.

In the NHS England PCDD, the rate of antipsychotic prescribing in people with dementia who do not have psychosis is 6.9% (35,217/510,536) compared to ADNeT's 6% (106/1774) and SveDem's 4.4% (254/5772) in residential care settings only. This higher rate likely reflects the NHS England data being based on prevalent cases, capturing patients who have progressed to moderate or severe dementia when behavioural and psychological symptoms requiring antipsychotic medication are more common, compared to the incident cases recorded in ADNeT and SveDem, where prescribing is assessed only at initial diagnosis when patients are more likely to be in the early stages of dementia. NAD‐MAS and NorCog do not report levels of antipsychotic prescribing. England's rate suggests that efforts over the last 20 years to reduce antipsychotic prescribing have been successful by global standards, but comparison with prevalent cohorts from other countries is needed to confirm this.

Registry data must be continually reviewed, and data collection decisions updated, to maintain relevance and impact. For example, in April 2025, the NHS England PCDD added tracking of patients with a dementia diagnosis who had also experienced delirium in the previous 12 months (4.2%, 21,317/510,536), enhancing monitoring of a comorbidity that is common in dementia and affects prognosis and management [16]. The currently recorded rate is likely an underestimate due to incomplete delirium ascertainment and coding [17].

The value of continuing to report the DDR in the NHS England PCDD warrants consideration. On the one hand, the DDR provides a simple and consistent metric of diagnostic coverage; however, it is hampered by limitations in its denominator. Estimated prevalence is based on the CFAS II study, which was conducted over 15 years ago, and is not adjusted for sociodemographic or economic factors such as ethnicity, deprivation and rurality, which are known to influence risk of dementia [18]. Consequently, apparent regional variations in DDR may reflect differences in population structure as opposed to true differences in dementia diagnostic performance.

The underlying driver for the DDR as a policy instrument was a concern that dementia was systematically underdiagnosed due to insufficient provision and cultural barriers to diagnosis among clinicians and the wider public. Indeed, the estimated DDR at the time of the then Prime Minister's Challenge on Dementia in 2012 was 42% [19]. Highlighting the ratio of diagnosed to undiagnosed cases was felt to be an effective lever for reducing such obstacles. However, this relies on the estimates of true prevalence being accurate. Given the inevitable changes in patterns of disease including dementia in the population over time, prevalence estimates based on CFAS II are becoming increasingly unreliable. Large scale community sampling to “refresh” the prevalence estimates would require significant funding. It is notable that health systems outside the UK do not judge their performance according to the ratio of diagnosed versus undiagnosed dementia cases.

The DDR had stabilised at just under 70% nationally for some years prior to the COVID‐19 pandemic at which time it dropped for reasons we previously reviewed [20]. It is now on a trajectory to return to what appears to be a similar natural level, reflecting the inevitable gap between prevalent and diagnosed cases for any slowly developing disease. Indeed, by 2018 the proportion of undiagnosed cases in dementia in England was estimated to be lower than for most other common long‐term conditions [21], suggesting that the specific causes of the low DDR in 2012 have been resolved. Further, assigning a high value to diagnosis per se risks overdiagnosis, a phenomenon that applies in dementia as in other conditions and is harmful to patients, families and wider society [22]. This does not of course mean that inequalities in access to diagnosis do not exist or should not be addressed [18].

## 3 Future Data Metrics

To more closely align with quality registries like SveDem, NorCog, and ADNeT, the NHS England PCDD could collect data on dementia severity at the time of diagnosis, categorised as mild, moderate or severe. However, this would require diagnostic services to record this and incentivisation of primary care providers to code the severity indicator in addition to the diagnosis. It is important to recognise that introducing any new coding requirements carries resource implications and would likely necessitate financial or contractual incentivisation. In the current fiscal climate, such expansion of data collection may be challenging. Automated inference of dementia severity based on already‐recorded clinical data would be preferable if the necessary algorithms were developed and validated.

An aspirational target might be to collect biomarker‐supported diagnosis data for Alzheimer's, as in SveDem and NorCog's use of CSF and blood biomarkers. Including such data could enhance comparability of data across registries and strengthen the evidence base for planning and resource allocation. However, this is likely to require linkage with secondary care records to an extent not currently feasible in the English NHS. Further, data on the proportion of people receiving National Institute for Health and Care Excellence (NICE) approved pharmacological interventions for dementia, including AD medications, as seen in SveDem and ADNet, which collect data on acetylcholinesterase inhibitor (AChEI) prescriptions would be a useful quality marker. Prescribing data is already contained in the primary care record but requires analytic resource to add to the currently published metrics. Other NICE‐approved interventions including cognitive stimulation therapy and carer psychoeducation, are not reported in the NHS England PCDD or other quality registries. Adding these would be challenging as GPs are not incentivised to record non‐pharmacological interventions and no code for carer psychoeducation is currently available. The development of integrated neighbourhood teams [23] may provide a mechanism to address this gap if such teams assume responsibility for dementia case management and routine recording of psychosocial interventions.

The Republic of Ireland recently launched a National Dementia Registry [24], prioritising quality indicators including the proportions of cases undergoing a basic dementia diagnosis workup, receiving specific pathological dementia diagnoses, and treated with antipsychotics, plus measures of quality of life for patients and carers, similar to SveDem's focus.

The European Brain Council's Rethinking Alzheimer's Disease white paper 2023 [25] identified relevant challenges, such as fragmented data collection, regional variations, lack of coordination between government levels, and absence of standardised diagnostic and data protocols. Several European countries are attempting to address these gaps by streamlining dementia data collection to form a European AD registry providing a standardised set of statistics [26]. This highlights the need to take a more standardised approach to aid direct global comparisons of dementia registry data.

## 4 What Matters to Service Users?

An important challenge is to identify data that capture what matters most to people with dementia and their carers. Although the number of people with a diagnosis and the DDR have increased steadily year on year since the COVID‐19 pandemic, these figures alone cannot provide sufficient insight into the quality, timeliness, or equity of care. NAD‐MAS data show that average waits from referral to diagnosis have lengthened from 124 days in 2021 to 151 days in 2023 [11]. Compared to other countries, where median waits for diagnosis are significantly shorter, England is falling behind [27]. Aligning memory service pathways with the 18‐week standard applied to most NHS outpatient care could offer a coherent and meaningful benchmark, as this addresses patient experience directly.

This paper has focused on data collected around the point of diagnosis and initial management, including provision of pharmacological and psychosocial interventions. However, building on the 10 Year Health Plan for England, longitudinal data on the quality of aftercare and post‐diagnostic support will be equally important [28].

## 5 Conclusions

High‐quality registries can provide the rich and systematic data needed to drive meaningful improvement in dementia care. By evolving the NHS England PCDD toward the standards set by international quality registries such as SveDem, NorCog, and ADNeT, England could promote cross‐country learning, and ensure that dementia services are evidence‐driven, equitable, and responsive to population needs.

Continued consideration is required to determine what constitute the most useful data for improving the quality of dementia in meaningful ways. Whatever the answer, dementia must remain a national priority. With over half a million people in England currently diagnosed, the challenge extends far beyond diagnosis to encompass lifelong care and support for individuals and families. It is not only the numbers that matter, but also the problems they are intended to solve and the outcomes they ultimately help deliver.

## Funding

RH is supported by University College London Hospitals' National Institute for Health Research (NIHR) Biomedical Research Centre. JH is supported by the UCL ADAPT study (Blood Biomarker Challenge) and an Alzheimer's Research UK Clinical Research Training Fellowship (CRTF2023B‐003).

## Ethics Statement

This was aggregate data provided by GP practices and processed by NHS England, and publicly available reports from registries including SveDem, ADNeT, NorCog, and a national audit report NAD‐MAS, without individual patient identifiers.

## Conflicts of Interest

JDI is NHS England National Clinical Director for Dementia and Older People’s Mental Health. Until April 2024 he was Clinical Director of NHS England (London) dementia clinical network. The other authors have no conflicts of interest to declare.

## Data Availability

Data sharing not applicable to this article as no datasets were generated or analysed during the current study.
